# Marked elevation of serum lactate dehydrogenase in primary myelofibrosis: clinical and prognostic correlates

**DOI:** 10.1038/s41408-017-0024-9

**Published:** 2017-12-18

**Authors:** Sahrish Shah, Mythri Mudireddy, Curtis A. Hanson, Rhett P. Ketterling, Naseema Gangat, Animesh Pardanani, Ayalew Tefferi

**Affiliations:** 1 Division of Hematology, Department of Internal and Laboratory Medicine, Mayo Clinic, Rochester, MN USA; 2 Division of Hematopathology, Department of Internal and Laboratory Medicine, Mayo Clinic, Rochester, MN USA

Primary myelofibrosis (PMF) is a clonal stem cell-derived myeloproliferative neoplasm (MPN), characterized by dysregulated proliferation of megakaryocytes, myeloid and erythroid cells, extramedullary hematopoiesis, and reactive changes in the bone marrow, including reticulin deposition, osteosclerosis, and neo-angiogenesis^1^. Patients with PMF often experience transfusion-dependent anemia, hepatosplenomegaly, vascular events, infections, cachexia, leukemic transformation, and shortened survival^1, 2^. About 90% of the patients with PMF carry *JAK2, CALR*, or *MPL* mutations, which are often mutually exclusive and are referred to as “driver” mutations^1, 3^. Current prognostication in PMF employs the International Prognostic Scoring System (IPSS)^4^, Dynamic IPSS (DIPSS)^5^, and DIPSS-plus^6^. Additional prognostic contribution from driver mutational status and presence or absence of high-risk mutations, such as *ASXL1* and *SRSF2*, has since been realized. Furthermore, the World health Organization (WHO) classification system underscores the prognostically relevant distinction between overt and early/prefibrotic PMF (pre-PMF)^7^. Most recently, we reported leukocytosis-independent contribution of serum lactate dehydrogenase (LDH) in essential thrombocythemia (ET)^8^. In the current study, we looked for a similar possibility in PMF, in the context of conventional risk models.

The study was approved by the Mayo foundation institutional review board. Diagnoses of overtly fibrotic PMF and pre-PMF were based on the 2016 WHO criteria^9^. Additional selection criteria included the availability of serum LDH at time of referral. Marked elevation of serum LDH was defined as a value of ≥1000 U/L (i.e., over fourfold increase from the upper limit of the normal range for our institution, which was 122–222 U/L), based on preliminary analysis of the threshold prognostic effect. Using conventional statistical methodology, univariate analysis identified several clinical and laboratory parameters that were associated with shortened survival. These parameters were then combined in a step-wise manner for multivariable analyses by using Cox proportional hazard regression model.

Among 357 study patients, 311 had overt PMF and 46 pre-PMF. The median serum LDH level for all patients was 514 U/L (range 136–2263), with a significant difference between overt PMF (532 U/L; range 136–2263) and pre-PMF (401 U/L; range 180–1237; *p* = 0.0003). In order to minimize confounding from this observed difference and considering the small number of patients with pre**-**PMF, further analysis was limited to the 311 patients with overt PMF (median age 64 years; 66% males); driver mutational status was *JAK2* in 66%, type 1/like *CALR* 16%, type 2/like *CALR* 4%, *MPL* 5% and triple-negative 9%. DIPSS-plus risk distribution was 31% high, 43% intermediate-2, 15% intermediate-1, and 12% low; 30% displayed red cell transfusion dependency and 37% abnormal karyotype, including 14% with unfavorable karyotype; thirty-seven (12%) patients displayed marked elevation of LDH (≥1000 U/L), which correlated with higher leukocyte count (*p* = 0.03), circulating blast percentage (*p* = 0.03), and *SRSF2* mutational frequency (44% vs. 12%; *p* < 0.0001).

**Fig. 1:**
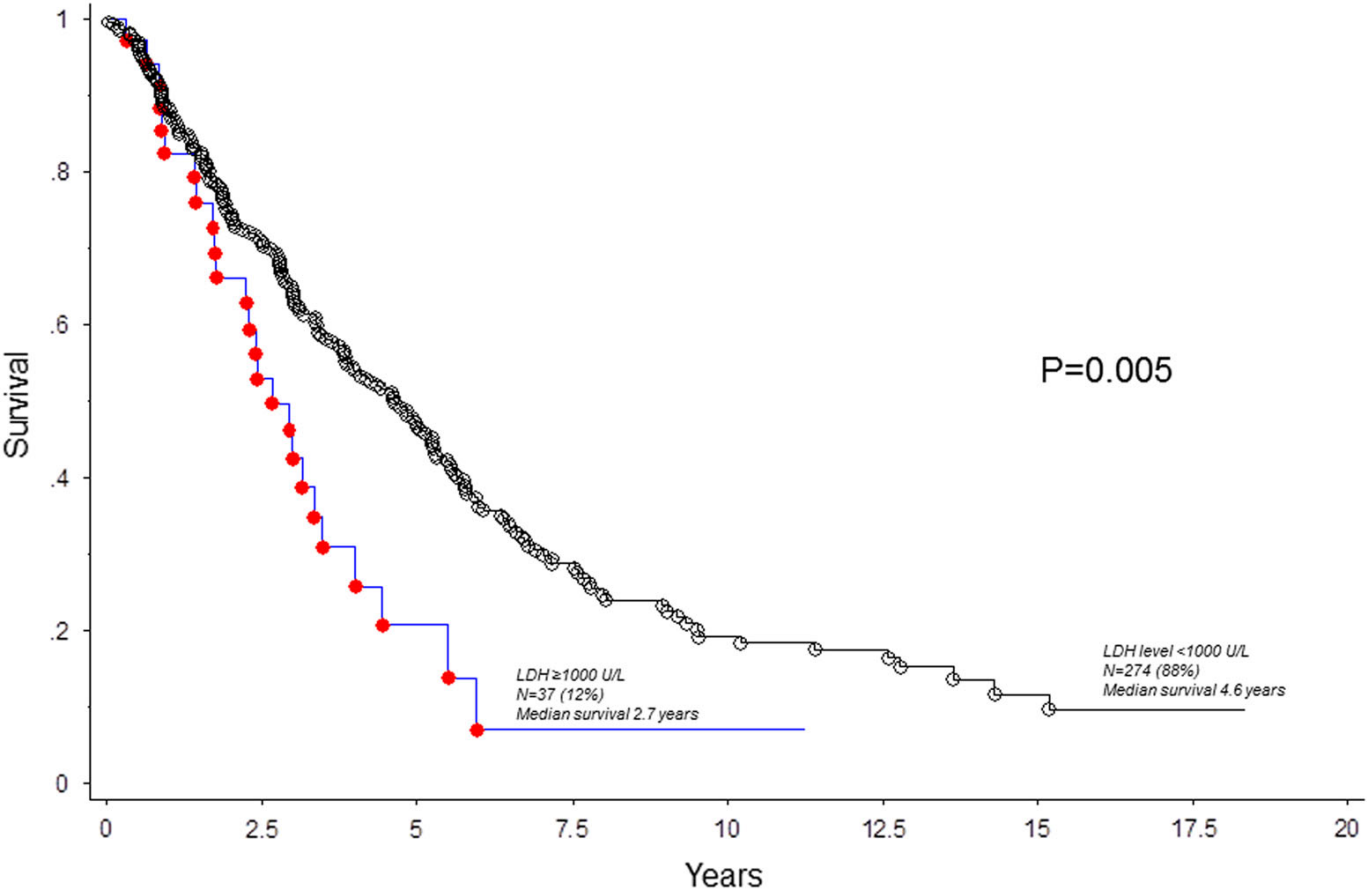
Survival in 311 patients with overtly fibrotic primary myelofibrosis, stratified by serum level of lactate dehydrogenase (LDH)

After a median follow-up of 3 years, 199 (64%) deaths and 31 (10%) leukemic transformations were documented. In univariate analysis, increased serum LDH level was associated with inferior survival, both as a continuous variable and as a categorical variable with the cutoff level of 1000 U/L (HR 2.20, 95% CI 1.3–3.1; *p* = 0.001); the adverse survival effect LDH ≥ 1000 U/L was independent of DIPSS-plus (HR 1.6, 95% CI 1.1–2.5; Table 1). Other variables that were significantly associated with shortened survival, on univariate analysis, included all eight DIPSS-plus variables (*p* ≤ 0.01 in all instances), absence of *CALR* type 1/like (*p* < 0.0001), and presence of *ASXL1* (*p* < 0.0001) or *SRSF2* (*p* = 0.0006) mutations (Table 1). In multivariable analysis that included only genetic risk factors, serum LDH retained its significance (HR 2.2, 95% CI 1.3–3.6), along with absence of *CALR* type 1/like, presence of *ASXL1* or *SRSF2* mutations, and unfavorable karyotype (Table 1). In multivariable analysis that included only clinical variables, serum LDH ≥ 1000 U/L was again independently predictive of shorter survival (HR 1.7, 95% CI 1.1–2.6), along with age >65 years, hemoglobin <10 g/dl, platelets <100 × 10^9^/l, leukocyte count >25 × 10^9^/l, and constitutional symptoms (Table 1). Patients with marked LDH elevation were also more likely to undergo leukemic transformation (HR 3.1, 95% CI 1.2–7.6). Figure 1 presents survival comparison stratified by serum LDH ≥1000 U/L: 2.7 vs. 4.6 years in the presence or absence of LDH ≥1000 U/L, respectively (*p* = 0.005).

**Table 1:** Univariate and multivariable analyses of survival in 311 patients with primary myelofibrosis, in whom information on serum level of lactate dehydrogenase, at time of presentation, was available

Variables	Univariate analysis			Multivariable analysis		
	Hazard ratio	95% Confidence interval	*P* value	Hazard ratio	95% Confidence interval	*P* value
LDH ≥1000 U/l	2.02	1.33–3.09	**<0.01**	3.25	1.87–5.65	**<0.01**
Age >65 years	2.33	1.74–3.11	**<0.01**	2.26	1.69–3.03	**<0.01**
Hemoglobin <10 g/dl	1.81	1.36–2.41	**<0.01**	1.73	1.30–2.31	**<0.01**
Leukocytes >25 × 10(9)/l	2.84	1.96–4.11	**<0.01**	2.28	1.55–3.34	**<0.01**
Platelets < 100 x 10(9)/l	1.75	1.28–2.40	**<0.01**	1.50	1.08–2.09	**0.014**
Circulating blasts ≥1%	1.44	1.08–1.91	**0.01**	1.16	0.86–1.58	0.3
Transfusion-dependent	1.87	1.38–2.52	**<0.01**	1.21	0.76–1.93	0.4
Constitutional symptoms	1.99	1.48–2.67	**<0.01**	1.75	1.29–2.38	**<0.01**
Unfavorable karyotype	1.61	1.08–2.40	**0.01**	1.69	1.11–2.57	**0.013**
DIPSS-plus risk stratification						
Intermediate-1	1.44	0.71–2.92	**<0.01**	1.24	0.59–2.62	0.56
Intermediate-2	3.49	1.91–6.37		2.39	1.08–5.31	**0.03**
High	6.84	3.71–12.58		2.98	0.98–9.01	0.05
Absence of *CALR* type 1/like	4.15	2.47–6.96	**<0.01**	3.05	1.77–5.27	**<0.01**
* ASXL1*-mutated	2.24	1.60–3.13	**<0.01**	2.39	1.63–3.49	**<0.01**
* SRSF2*-mutated	2.08	1.37–3.15	**<0.01**	1.00	0.61–1.65	0.97

*LDH* lactate dehydrogenase, *DIPSS* Dynamic International Prognostic Scoring System

Bold values indicate statistically significant differences

The current study suggests that marked elevation of serum LDH independently predicts shorter overall and leukemia-free survival in PMF. In this regard, our observations are similar to those recently communicated for ET^8^. In both instances, the prognostic contribution from serum LDH was independent of and possibly superior to that of leukocytosis, which is otherwise known to be directly correlated with serum LDH level. We believe that these observations are indicative of the possibility that serum LDH level is a biologically more accurate marker of cell turnover, and thus degree of clonal myeloproliferation. Other studies have also looked into the diagnostic role of serum LDH in MPN^9–13^. However, diagnostic utility is limited by the considerable overlap of readings among patients with distinct MPN categories^14^, although its discriminatory value in distinguishing pre-PMF from ET has been stressed^15^. Whether or not serum LDH level is to be included in future prognostic models for PMF or ET requires additional studies that take into account the confounding effect from other comorbidities that are known to be associated with increased serum LDH, such as liver disease.
